# An ethnography study exploring factors that influence social isolation in care home residents living with dementia and hearing loss

**DOI:** 10.1186/s12877-023-04296-0

**Published:** 2023-09-25

**Authors:** Nisha Dhanda, Helen Pryce

**Affiliations:** 1 Institute of Applied Health Research, University of Birmingham, Birmingham, UK; 2 University of Birmingham Dubai, Dubai International Academic City, PO Box 341799, United Arab Emirates; 3 College of Health and Life Sciences, Aston University, Birmingham, UK

**Keywords:** Communication, Social isolation, Dementia, Hearing, Ethnography

## Abstract

**Background:**

Hearing loss and dementia are highly prevalent conditions amongst older adults living in residential care. The consequences of living with these conditions may include social withdrawal and reduced communication opportunities. We sought to examine patterns of communication and interaction in residential care and explore resident, staff, and relative perspectives within two care homes located in Birmingham, UK. This enabled an understanding of how communication environments contributed to social isolation.

**Methods:**

This work used ethnography methodology to explore mechanisms that created and maintained social isolation in older adults living with dementia and hearing loss. A planning and engagement phase took place in four care homes. This was followed by an environmental audit, observations, and interviews. Data generated were analysed using Grounded Theory methods.

**Results:**

There were 33 participants (16 residents, 11 care staff, and six relatives) who took part in the observations and interviews. Residents experienced social isolation through lack of meaningful conversation with others and being misunderstood. Additionally, observations of residents’ interactions informed the overall findings. A Grounded Theory model was employed to explain the core phenomenon of social isolation. The main contributors were internal and external barriers to communication, and reduced opportunities for meaningful conversation.

**Conclusions:**

There is a wide range of social isolation that care home residents experience. This was not always associated with the severity of hearing loss but rather communication ability. Simple interventions such as staff dining with residents and focussing on improving communication could reduce social isolation within residential care settings.

**Supplementary Information:**

The online version contains supplementary material available at 10.1186/s12877-023-04296-0.

## Introduction

### Overview of topic area

Residential care settings in the UK currently house 39% of the 750,000 adults over 65 living with dementia [1]. The prevalence of dementia or mild cognitive impairment in residential or nursing homes can be as high as 80%, according to statistics reported by Alzheimer’s Society [2]. Undiagnosed hearing impairment in individuals residing in care is as high as 90% [3]. There is a complex relationship between hearing loss and dementia or cognitive impairment in older adults [4]. Associations between the two have been demonstrated in epidemiology data [5–7], but definitive mechanisms are yet to be established. When these conditions co-occur, there is a change in people’s communication patterns and abilities, and an increased likelihood of social withdrawal and isolation [8]. Managing hearing impairment and dementia within a care home environment can be challenging for residents [9], which can inevitably lead to, or exacerbate, the experience of social isolation [10].

A policy position paper from the Alzheimer Society of Ireland [11] highlighted the issue of loneliness among people with dementia and the negative impact it can have on their well-being. The paper outlined the importance of tackling loneliness among people with dementia and urgency of foundational research for intervention development to support older adults living with dementia, and associated comorbidities. This ethnographic study offers valuable insights and understanding regarding the factors contributing to social isolation, thereby informing the design of interventions aimed at mitigating the risk of loneliness.

### Past and present of residential care

Residential care in England has a complex past and present. The concerns highlighted by Townsend’s 1962 [12] study on residential care provision in the 1960s continue to be felt and observed. Specifically, that some communal homes “do not adequately meet the physical, psychological and social needs of the elderly people living in them” [13] Residential and nursing care facilities are hugely complex structures. The pressure on care facilities is rapidly rising as the number of older adults leaving hospital and discharged into nursing homes is increasing [14]. Therefore, the current state of care home provision is stretched. Some recommendations for improvement have included funds to be set aside for the training and resources of care facility staff so that they are equipped to support individuals with complex requirements and a history of frailty; easy access to NHS funded rehabilitation and outpatient services; and a defined and standardised multidisciplinary team for residents [15].

### Communication in residential care

In residential settings, interactions between carers and residents are essential [16]. Care staff can effectively communicate each resident’s needs and provide individualised care [17]. Even though communication is important in the delivery of care, both residents and carers frequently express dissatisfaction with this component of care [18]. Residents often feel disempowered, dehumanised, and undervalued because they believe the carers are not readily available to address their concerns or fulfil their demands [19]. For several reasons, communication is difficult in residential care facilities. First, despite some care personnel regularly overlooking them, communication issues, such as hearing impairment, are widespread [20]. According to earlier studies, 70% of residents failed two or more communication screening tests, with hearing and cognitive impairment accounting for many of these failures [21]. Despite the significant prevalence of hearing impairment in these environments, it is essential to prevent workers from underestimating residents’ hearing impairment, which impacts communication and the standard of care [22].

The physical environment and construction of residential care settings are frequently suboptimal for communication, with reports of excessive noise and reverberation levels [23]. It is commonly known that hearing loss and difficulties hearing in noisy environments correlate. These spaces typically consist of hard surfaces, which create unfavourable acoustics, high noise levels, and reverberation [24], all of which hinder communication involving hearing. Bright surfaces usually worsen the auditory environment, making it harder for residents to employ visual cues and limiting their communication ability [25]. High noise levels may also negatively affect people living with dementia, such as causing increased agitation [26].

### Defining social isolation

The National Academies Sciences Engineering Medicine consensus study report (page 1) [27] define social isolation as: “the objective state of having few social relationships or infrequent social contact with others”. Therefore, the emphasis is on the number of social connections rather than the quality of relationships, engagement, and interactions. This differs from our interpretation of social isolation. We view social isolation through the social identities approach, derived from group belonging that affects health and wellbeing [28]. The basis of this approach is that the way you see yourself is informed by the nature of the social groups that you’re in, as first described by Tajfel [29]. So, a person’s defined ‘sense of self is primarily influenced by their membership in social groups rather than their ‘personal’ identity [30]. A person’s sense of self is formed and maintained in part by attributes that they share with others and where they can relate to a sense of common experience [29]. We have evolved to live in social groups, giving us a sense of direction and purpose [31]. Social identity is the core of group behaviour and can be deemed essential for us to thrive (when group participation is positive) [32]. When this is applied to health and wellness, the identification hypothesis states that a person will experience health-related benefits depending on their group membership and the amount they identify within that group [33]. Care homes offer social environments for people with similar demographic characteristics [32]. Therefore, we used Tajfel’s [29] social identity approach as a pre-existing assumption in understanding the social groups and interactions within the home. Tajfel [29] referred to the change from ‘I’ to ‘we’ in self-identification when individuals feel part of a positive social group experience. Our pre-existing assumption specifically relates to how social identity approach can influence residents’ interactions with others, and how positive intergroup relations can be promoted to help reduce the occurrence and maintenance of social isolation [33].

Whilst many researchers have recognised the value and importance of the social identity approach in terms of the interactions within social groups [34–36], what’s been missed is the amount of meaningful engagement and identification within those groups. We, therefore, refer to social isolation as the degree of social connectedness and meaningful engagement between individuals within their social context.

### Hearing loss and social isolation

Hearing loss can worsen actual or perceived social isolation [37], leading to a reduced number of social networks and the quality of social connections [38]. Active management of hearing loss may reduce social isolation as long as an individual has a sense of identity within a group [39]. Therefore, the frequency of contact is less important than the quality of social interactions and the significance of one’s social identity within the group. When hearing loss is combined with cognitive impairment, the absence of social identity may perpetuate social isolation [40]. This is especially so where social isolation exists as both an outcome and moderator of worsening cognitive function [20, 41].

There is a lack of evidence in care homes exploring the complexities of hearing loss, social isolation, and dementia in older adults. Previous studies have emphasised the impact of social and environmental elements on effective communication among people [20, 42]. Even though using hearing aids does not usually improve social interaction [42], there are communication hurdles since there is insufficient staff training on sensory impairment [43]. To improve residents’ overall well-being in a compassionate environment, it is crucial to understand their needs, wants, and lived experiences [44]. Specifically, regardless of cognitive capacity, connectivity and social engagement were crucial for residents’ quality of life [45]. A study of residents’ perceptions of the care home as their home revealed a desire for meaningful relationships within their surroundings [46]. This would enable them to thrive and overcome their feelings of homesickness. This is further supported by the importance of having a private area for residents and visiting spouses to connect and maintain their relationships within a home to sustain meaningful connections [47].

Communication between care personnel and residents is essential for high-quality care to be provided, especially in residential settings where carers are frequently the only source of social engagement with residents [48]. Therefore, effective interpersonal communication skills that are adapted to residents’ communication difficulties are crucial for care providers working in these settings [49]. According to Kerr et al. [50], interventions to enhance the communication abilities of care professionals should be multidimensional and include the following three elements: practise, support, and educational training. Empowering training techniques that are highly interactive, learner-centred, and didactic can be effectively employed.

A realist synthesis of hearing-related communication in care homes revealed several context-specific factors that would optimise communication for individuals living with hearing loss and dementia [51]. Staff training to better understand residents’ needs and to ‘know the person’ were important in the occurrence of meaningful communication. It is essential to understand the mechanisms that perpetuate social isolation. The Medical Research Council framework [52] on developing and evaluating complex interventions recommends a clear understanding of mechanisms and active ingredients before intervention development. This increases the chances of effective and appropriate interventions for the specific health condition and population [53].

### Rationale for study

To focus on this context-specific feasibility and acceptability, we adopted realist thinking to determine “what works, for whom, and in what circumstances” [54] to provide an understanding of the causal mechanisms underpinning social isolation in residential care settings. The use of realist thinking is important in this study because it is informed by closed system thinking i.e., there is consideration and acknowledgement that the social structures and mechanisms, are operating in a complex system that is “closed” because residents are constantly within the physical space of the home [55]. Therefore, we rely on the context, mechanisms, and outcomes to understand the closed system within the overall ethnography. This study explored the mechanisms of how and why social isolation occurs amongst individuals living with self-reported (with or without a formal diagnosis) hearing impairment and cognitive impairment in two residential care settings. In keeping with the social identity approach to health and wellbeing, our starting position is that social engagement and social connectedness between individuals is the core component of meaningful relationships. This is the focus of our study and the phenomenon that we have set out to explore.

## Materials and methods

### Design

We used ethnography methodology to explore the mechanisms of social isolation based on participants’ lived experiences. Here we considered examples of social isolation to include observations and descriptions of resident participants having a degree of connectedness in conversation, relationships, and contentment within their environment. Realist research aims to determine how the context, mechanisms, and outcomes of a phenomenon can help to explain its social construction [54]. The phenomenon of interest was social isolation. Grounded Theory methods and analyses were complemented with realist informed thinking to determine the mechanisms underpinning social isolation. This allowed the development of data-driven theory within a context-specific framework [56].

**Fig. 1:**
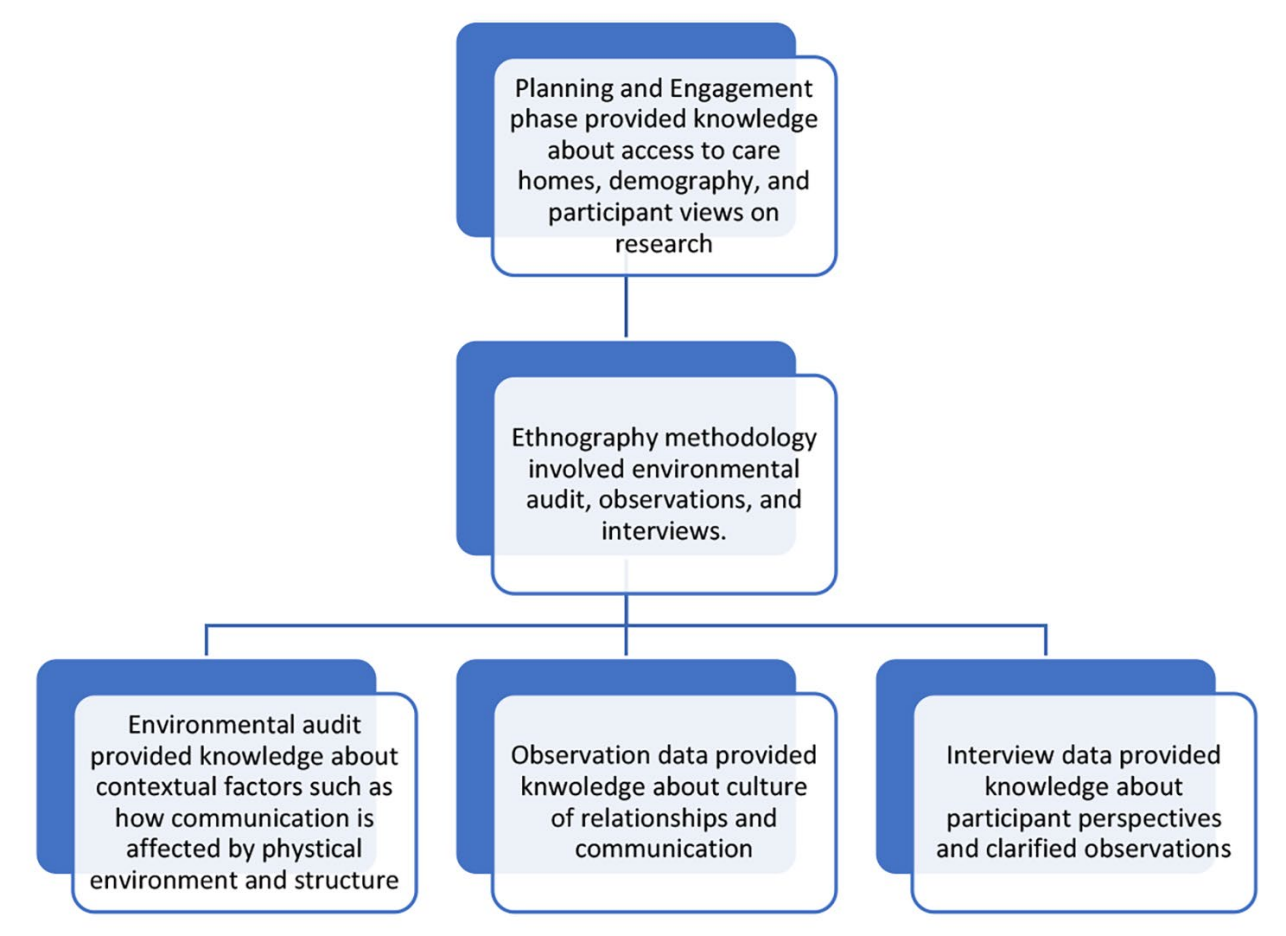
A diagram outlining the design components of the study and their relationship to one another. The richness of data generated from an ethnography methodology can provide a meaningful interpretation and explanation of social isolation mechanisms within a care home setting

An initial Planning and Engagement (PaE) phase aimed to establish the values and access that would influence research conduct and determine feasibility (see Fig. 1).

The ethnographic empirical work consisted of an environmental audit, observations, and semi-structured interviews. The environmental audit facilitated a description of relevant contextual features. Participant observations and interviews were conducted using critical ethnographic methods [57]. This involved identifying mechanisms and outcomes about the culture of communication and inclusion within the homes for underrepresented groups. The formal interviews provided insights into perspectives of the community, including residents, staff, and family members, and contributed to the identification of mechanisms and outcomes. This pluralist approach allowed for data triangulation, informed by realist thinking [58]. The findings presented in this paper reflect the views of the resident, staff, and relative participants.

### PaE findings

The data gathered during the PaE phase was used to determine the feasibility and appropriateness of each care home. Through discussion and understanding of the care home routines and schedules, practical issues were resolved. This included staff requesting that observations not be disruptive to their duties and for interviews at the beginning or end of shifts. Additionally, observations and interviews were only in communal areas due to safeguarding policies. This phase allowed us to become a familiar presence to staff and residents. As a result, we integrated data gathering into the community as frequent visitors. This allowed staff and residents to feel relaxed in the researcher’s presence.

The ethnographic work was conducted in two of the four homes involved in the initial PaE phase. They were part of the same private company and specialised in Dementia care. Care home A was in a deprived area of Birmingham, categorised within urban adversity. According to Acorn postcode profiling, care home B was in a more affluent area, classified within comfortable communities [59]. Each home catered for up to 40 residents living with or without dementia who require personal or nursing care.

### Sampling and participants

Approximately 160 people were living with dementia across the four care homes involved in PaE, and 100 of these persons could consent at any one time according to care staff assumptions about participants’ mental capacity. A report evaluating a network of research-ready care homes found that recruitment rates in residential care account for approximately one-third of all potential residents eligible for participation [60]. Therefore, 30 participants out of a potential 100 was deemed a reasonable number for recruitment to ensure sufficient variation, with approximately 7–8 participants from each home, if every home chose to participate in the ethnographic research. The aim was to provide a sample large enough to cover age, gender, and ethnic variations, using purposive sampling methods, within the realistic recruitment rate of 30 participants across the four homes [60]. A priori maximum variation could not be achieved because we excluded participants without capacity to consent. Nonetheless, the contrasting postcodes of the homes allowed socioeconomic variations between participants, and within each home we sought to recruit a diverse sample of participants via purposive sampling. The final sample included participants from only two of the four homes because of the COVID-19 restrictions that were in place during March 2020.

### Ethical considerations

Care staff assisted us in identifying suitable resident participants based on mental capacity. We had undertaken training on the Mental Capacity Act (2005) [61] before study commencement. Residents who could consent at any one time could participate in the study observations and interviews. It could be deemed unethical to include participants who cannot agree and require a proxy decision-maker, whereas those with capacity are available [62].

Of the 50 eligible residents across both homes, 16 had consented to take part and could provide fully informed consent at any one time. This figure aligned with Davis et al. [60] who suggested that one-third of eligible participants in care homes are likely to participate for the whole duration of the study. Due to fluctuations in capacity, verbal consent was taken at each interaction with willing residents to ensure ethical research standards were upheld and residents understood the research’s nature and involvement. Residents were approached directly and informed about the purpose of the study. Eleven staff members and six relatives consented to participate and were approached directly by us. Levels of participation involved observation, interview, or both. Field notes were only recorded for participants who had consented to participate in the research. These were written by hand, during or immediately after observations. All participants were given pseudonyms to ensure anonymity.

This study was given ethical approval by the Health Research Authority, West Midlands - Coventry & Warwickshire Research Ethics Committee. Participants provided fully informed consent before taking part. The informed consent covered both the observations and interviews for residents, staff, and relatives. Participants were informed that they could opt out of one or the other or withdraw completely at any time without reason.

### Data Collection

The environmental audit consisted of a proforma template with sections on lighting, audio and sound resources, echo, noise reduction, and internal/external noises (see appendix 1). The multiple visits to each home during the PaE phase highlighted the physical features and structures of each home, which informed the components of the proforma. In addition, an expert in care home research, who was consulted during the PaE phase, assisted with the development of the proforma.

On the first day of data collection at each home, ND walked through the communal areas of each home (lounges, dining room, main corridors) and completed the proforma, which provided information on the context of each home used to support the realist framework. This determined the approach for subsequent activities within the research and enriched the interpretation of observations and interview transcripts about environmental factors that contributed to the presence of social isolation. The environmental audit was completed during daytime hours when most residents were seated in the communal lounges, and care staff were busy with their daily tasks.

Ethnographic observations were conducted using pen and paper to record field notes, and an encrypted Dictaphone was used for audio interview recordings. We spent two weeks in each home, visiting on average for four hours a day between 7am-7pm. This allowed daytime routine, mealtimes, and structured activities to be observed. This is where most communication interactions occur, as evidenced by the PaE phase. Therefore, it was an ideal opportunity to capture the types of exchanges between residents, between residents and staff, and when relatives were most likely to visit. There was consideration to how residents used the space and furniture, how furniture positioning inhibited and facilitated interactions, and how residents interacted with staff and family members. Observations took place in communal areas of the homes only, such as dining rooms, lounges and libraries.

Interviews took place following observation and informal discussion with participants. Three separate interview schedules were produced to represent the different focuses of the three participant groups. The questions covered topics on current activities carried out within the home; communication experiences of residents with other residents and with care staff; ideas for minimising social isolation in the home; factors that contribute to social isolation; listening environment of the home; access to sound and conversation; opportunities for social interaction. The interviews were designed to be semi-structured, and therefore the questions listed on the interview schedules were used purely as a prompt rather than a script.

The constant comparative method of data collection was used [63]. This meant that the interview questions were informed by environmental audit findings and ethnographic observations. The interview responses then informed further observations of specific activities or events within the homes. Therefore, an iterative approach to data collection and analysis supported production of the Grounded Theory model.

### Data Analysis

Data analysis of field notes and audio interview recordings were informed by grounded theory [64]. Interviews were transcribed verbatim. An iterative data gathering and analysis process as part of a theoretical sampling framework [65]. This analysis aimed to create a model that explains a multidimensional dynamic theory of how different factors affect human behaviour in residential care settings.

Coding was completed line-by-line to maximise initial descriptive categories that later became analytic themes [66]. Three stages of coding took place: open coding, axial coding, and selective coding. The former involved examining, comparing, conceptualising, and categorising data. This was conducted for individual data frameworks, i.e., all residents, care staff, relative interview and observation data, and collective data frameworks. This allowed data to be analysed in its own right and compared to other data within the project. Axial coding involved reassembling data into groupings based on identified relationships, patterns, and themes. Selective coding describes the central phenomenon and underlying mechanisms [67].

Data analysis was completed using NVivo software. Data analysis ceased upon completion of selective coding when repetitions in mechanisms across both research sites became apparent. Through multiple verbal discussions, key concepts that supported the central phenomenon of social isolation mechanisms were labelled by ND and checked by HP to assist the development of the final model. The data were analysed using pre-existing assumptions of the communal nature of care home settings, informed by social identity approach for health [29]. The researchers were interested in observing examples of social group roles and interactions to understand the context, mechanisms, and outcomes of how and why social isolation may occur in such settings.

## Results

There were 33 participants (16 residents, 11 care staff, and 6 relatives) included in the ethnography across two care homes. The two care homes were located in contrasting socioeconomic locations within Birmingham, UK. Care home A was located in a less affluent area than care home B. There was an almost equal number of participants included from each home. All resident participants had some degree of hearing impairment and dementia. The staff participants’ length of experience ranged from six months to over ten years. The roles of staff participants included care assistant, apprentice, team leader, housekeeper, care home manager, handyman. Tables 1, 2 and 3 provide descriptions of the resident, staff, and relative participants at care homes A and B.

**Table 1:** Description of resident participants

Participant Pseudonym (care home A or B)	Age-band and Gender	Description*	Interview Details	Level of Participation
Wendy (A)	90–95, F	Early-stage dementia, mild hearing loss.	Interviews took place in the dining room before lunch.	Observation and Interview
Lana (A)	80–85, F	Sings quietly to herself, observes everyone. Moderate-stage dementia, moderate hearing loss.	Interviews took place in the dining room before lunch.	Observation and Interview
Emily (A)	85–90, F	Petite lady often sits on the edge of the chair, mutters to herself a lot. Moderate-stage dementia, mild hearing loss.	Interviews took place in the dining room before lunch.	Observation and Interview
Gia (A)	80–85, F	Often walking around on Zimmer frame talking to staff and other residents. Moderate-stage dementia, moderate hearing loss.	Interviews took place in library area before and after lunch.	Observation and Interview
Vina (A)	75–80, F	Smiles and waves at others but do not speak often. Early-stage dementia, moderate hearing loss.	Interviews took place in library area before and after lunch.	Observation and Interview
Cilla (A)	70–75, F	Laughs often. Moderate-stage dementia, moderate hearing loss.	Interviews took place in dining room after lunch.	Observation and Interview
Kelly (A)	75–80, F	Always sits with her coat on and handbag on shoulder. Moderate-stage dementia, mild hearing loss.	Interviews took place in library area before and after lunch.	Observation and Interview
Leon (A)	75–80, M	Quiet, spends most of his day sitting in the entrance area of the home looking outside. Moderate-stage dementia, moderate hearing loss.	Interviews took place in entrance area after lunch.	Observation and Interview
Penny (B)	75–80, F	Always smiling and sitting next to Chloe or Susan. Early-stage dementia, moderate hearing loss.	Interviews took place in quiet lounge after lunch.	Observation and Interview
James (B)	80–85, M	Quiet, likes to sit by himself in dining room or upstairs lounge. Moderate-stage dementia, severe hearing loss.	N/A	Observation
Chloe (B)	70–75, F	Husband also resides in care home. Very interested in staff members. Early-stage dementia, mild hearing loss.	Interviews took place in dining room before lunch.	Observation and Interview
Sienna (B)	70–75, F	Only comes down to communal areas for mealtimes and activities. Early-stage dementia, mild hearing loss.	Interviews took place in dining room before lunch.	Observation and Interview
Jenny (B)	75–80, F	Quiet, recently had a fall. Early-stage dementia, moderate hearing loss.	Interviews took place in ground floor lounge after lunch.	Observation and Interview
Miranda (B)	75–80, F	Laughs often. Wore two hearing aids. Early-stage dementia, severe hearing loss.	Interviews took place in quiet lounge after lunch.	Observation and Interview
Desmond (B)	75–80, M	Very talkative, described the home as his place of work. Moderate-stage dementia, mild hearing loss.	Interviews took place in dining room before lunch.	Observation and Interview
Susan (B)	70–75, F	Quiet, always smiling. Nervous nature. Early-stage dementia, moderate hearing loss.	Interviews took places in quiet lounge before lunch.	Observation and Interview

*Hearing loss descriptions are based on my clinical observations and experience of how well a person can hear and communicate

**Table 2:** Description of staff participants

Participant Pseudonym	Age-band and Gender	Description	Interview Details	Level of Participation
Jane (A)	40–45, F	Care Assistant. Talkative to residents and other members of staff.	Interviews took place in dining room before lunch.	Observation and Interview
Lucy (A)	30–35, F	Apprentice. Spends a lot of time with Lottie.	Interviews took place in dining room before lunch.	Observation and Interview
Sarah (A)	30–35, F	Team Leader. Quiet, spends a lot of time sorting medications.	Interviews took place in dining room before lunch.	Observation and Interview
Delia (A)	40–45, F	Housekeeper. Spends a lot of time in resident flats having one-to-one interaction.	Interviews took place in dining room before lunch.	Observation and Interview
Lottie (A)	45–50, F	Care Assistant. Knows all the residents very well. Worked for 10 + years in home.	Interviews took place in dining room before lunch.	Observation and Interview
Ria (A)	45–50, F	Care Assistant. Quiet, spends a lot of time with Jane.	Interviews took place in dining room before lunch.	Observation and Interview
Helen (B)	25–30, F	Team Leader. Always walking fast up and down care home. Spends a lot of time doing medications.	Interviews took place in staff room during lunch.	Observation and Interview
Mike (B)	45–50, M	Handyman. Always present in communal areas of home. Knows all residents very well.	Interviews took place in dining room before lunch.	Observation and Interview
Alana (B)	40–45, F	Care Home Manager. Spends majority of time in her office upstairs but does join in with activities/tidying.	Interview took place in office before lunch.	Observation and Interview
Tasia (B)	25–30, F	Team Leader. Always busy and trying to interact with residents.	Interview took place in quiet lounge area during lunch.	Observation and Interview
Kim (B)	40–45, F	Housekeeper. Spends majority of time in resident flats but does try to interact as much as possible.	Interviews took place in dining room before lunch.	Observation and Interview

**Table 3:** Description of relative participants

Participant Pseudonym	Age-band and Gender	Description	Interview Details	Level of Participation
Martin (A)	60–65, M	Wendy’s far-distant relative. Visits once a fortnight.	Interview took place in entrance area during lunch.	Observation and Interview
Sylvia (A)	60–65, F	Wendy’s far-distant relative. Visits once a fortnight.	Interview took place in entrance area during lunch.	Observation and Interview
Greg (A)	30–35, M	Visits Mother once a fortnight. Attended with his daughter.	Interview took place in dining room after lunch.	Interview
Robert (A)	60–65, M	Cilla’s brother. Visits once a fortnight.	Interview took place in dining room after lunch.	Observation and Interview
Catrina (B)	85–90, F	Visits husband every day. Very active woman, keen to do as much as possible to care for husband.	Interview took place in Catrina’s husband’s room during lunch.	Observation and Interview
Yulanda (B)	35–40, F	Penny’s daughter. Visits 3–4 times a week.	Interview took place in quiet lounge area during lunch.	Observation and Interview

The environmental audit reported on each home’s physical features and structures. Table 4 provides a summary of the environmental audit results. In care home A the communal lounge was located next to the dining area with the door always open. This meant that meal and dining preparations could be heard from the communal lounge, in addition to other noise sources (television, radio, and Alexa device). By contrast in care home B, the communal lounge was located at the end of the corridor away from the dining room and loud noise sources. There were no sound resources identified in either home. For example, no telecoil loop system, no central speaker system, and no flashing or vibrating safety equipment to support hearing impaired people in emergencies. Both homes were part of the same business group so this could have been exclusive to this company, or a universal issue in care homes. The furniture choice in both homes was ideal for preventing or reducing echo within the communal areas. Care home B had an appropriate layout of furniture, conducive to encouraging conversation and interaction between residents. However, the smaller size of the communal area, compared to care home A, meant that relatives and friends visiting residents had little privacy. There were other communal areas and a library that offered alternative areas for privacy, however.

**Table 4:** Results of environmental audit at care homes A and B

	Care Home A	Care Home B
Main Communal Lounge – General Overview	Challenging listening environment. Television and radio often switched on simultaneously (and loudly) in different corners of the open plan space. High ceilings and carpeted floors. Located next to dining hall with door always open. Windows looking out to garden area.	Smaller than care home A. Carpeted floors and located at the end of a corridor. Television is the only sound source within this space. Located down the corridor from dining hall, windows looking out to front carpark.
Sound Resources	No sound resources identified such as telecoil for hearing aid input.	No sound resources identified such as telecoil for hearing aid input.
Furniture	Soft furnishings that prevented echo. Armchairs were placed in clusters of three or four.	Armchairs placed around the edge of the room facing inwards towards television.
Dining Room – General Overview	Hard floors and high ceilings, with limited acoustic absorption around the room. Sound of food preparation, crockery and cutlery from the kitchen was heard prominently.	Natural light, low ceilings and lino floors. Very little sound heard from the kitchen when sitting on the dining tables.

The dining area of care home A was very much like the communal area. In other words, high ceilings hard floors, and very little sound absorption. The dining area of care home B was smaller and quieter, which may have promoted more conversation between residents during mealtimes. However, there was a tendency for more communicative and mobile residents to eat their meals before those who were frailer and generally sleepy. This may therefore not be a fair comparison of mealtimes between the two homes.

### Development of Model

**Fig. 2:**
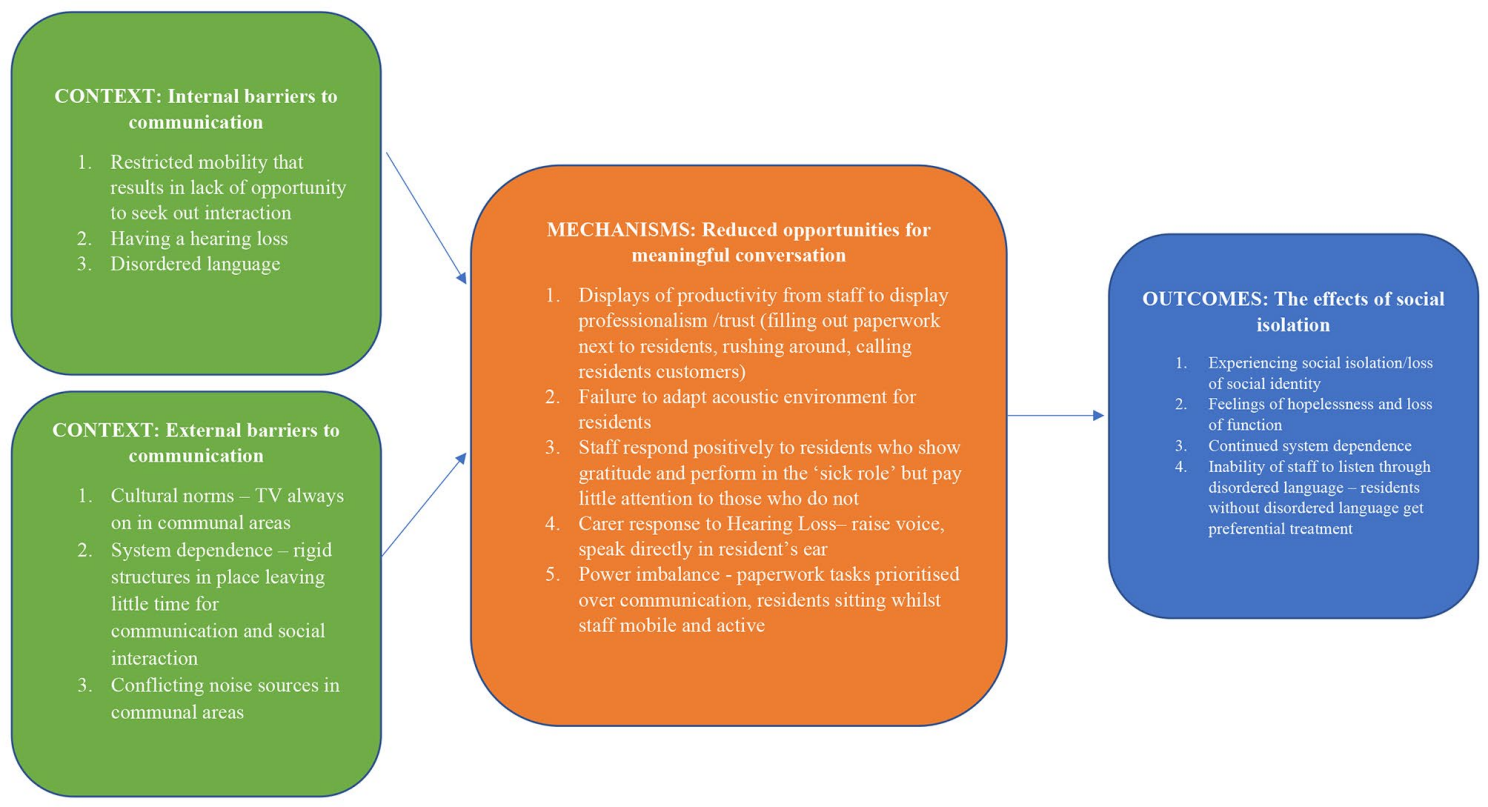
Summary model to explain the factors involved in the phenomenon of social isolation

The environmental audit and observations provided data on the context and strategies used by care staff. The interviews contributed to the context in greater depth, enabling the contextualisation of the data [75]. This led to identifying social isolation as a central phenomenon underpinned by internal and external communication barriers. Despite the differences in location and socio-economic status of the two homes, there were very few differences in the types of communication behaviours observed. Social isolation was fuelled by staff time constraints and the priority of physical tasks. Therefore, communication and meaningful conversation were not prioritised, leading to social isolation. The context, mechanisms, and outcomes of how and why this occurred are detailed. The role of the resident and staff participants within the structure of the residential care settings guided the analysis and development of the model (see Fig. 2).

### Context: Internal and external barriers to communication

Restricted mobility was categorised as a potential internal barrier to communication because most participants used walking aids to help them mobilise around the home. When they were on their feet, their pace was slow, and they often required assistance from a staff member to help them get from one place to another. Their motivation for moving around may have been reduced because of the additional support required, leading to a barrier for communication and interaction with other residents who were sitting on the other side of the communal lounge or dining area. The social identity approach suggests that a resident’s mobility could influence their social group role within the communal spaces of the home. A mobile and active resident may have a greater sense of belonging compared to a less mobile resident and may be able to conceptualise the “us” and “we” sentiment.

The presence of a hearing loss was observed as a possible internal barrier to communication. The hearing loss may have been exacerbated by the noisy conditions within communal areas of the home. High ceilings and hard floors provided poor acoustics, as evidenced from the environmental audit. In addition, competing sound sources from the television, radio and kitchen may have created a muffled listening environment for residents. With only one participant using hearing aids (Miranda), but all having some degree of hearing loss, the knowledge and awareness of hearing and communication management from staff appeared limited. Their response to those with hearing loss was met with gestures such as raising their voices and talking into the residents’ ears. Staff reported that the links with local Audiology services were virtually non-existent. Unlike optometry and podiatry who visited the homes regularly to screen residents, no such service existed for hearing health check-ups.

Disordered language was observed as another potential internal barrier to communication. The authors refer to disordered language where they observed residents with difficulties expressing meaning and nuance to other people through their speech. 10 of the 16 resident participants displayed examples of disordered language. The amount of disordered language a resident had appeared to be correlated with the severity of their dementia, and subsequently the amount of social isolation they experienced. For example, there was one resident (James) whom staff were very surprised agreed to participate in the study because they rarely spoke or interacted with anyone. When they were given undivided attention by the researcher and afforded the time to find their words, they conversed at length. Some words and utterances were unclear, but the researcher was able to piece together the sentiment based on their hand gestures and non-verbal cues. Therefore, the resident’s level of expressive language was observed to be a contributing factor to their level of engagement with others, and perhaps feelings of being socially isolation. This was striking because it appeared that the level of hearing impairment a person had was not a crucial factor, but rather the severity of dementia as this was the cause of the disordered language. This was further evidenced by observations of one participant (Miranda) with moderate-severe levels of hearing impairment and early-stage dementia having interactive and humorous conversations with staff members. This occurred across the room from another participant Desmond who had mild hearing impairment but severe dementia and disordered language. There was very little interaction between care staff and Desmond observed, apart from basic questions related to his physical care. During a conversation with the researcher, Desmond turned to a staff participant (Helen) and said “I can’t believe…this one (pointing at researcher)…so young and all this writing…what shall we do when the clouds break…(turns to researcher)…what did you say dear.” Helen smiled at Desmond and said “oh what are you like”, and then walked away. There was little effort made to understand what Desmond was trying to say or how he felt in that moment.

There were very clear routines and schedules within the homes that staff tried not to deviate from, indicating a strong dependence on the “care home system”. Staff were very much task-driven during their shifts and, therefore, appeared to consider activities or conversations with residents to be bonus tasks, only to be completed if everything else has gone smoothly and on time. The system dependence appeared to create an external barrier to communication. One resident became anxious when I asked if I could interview her in the dining room before lunch. She said, “Oh I do hope that’s okay I wouldn’t want to disturb anyone or interfere I know it’s dinner soon.” Therefore, it appeared as though residents connected more to the processes that occur within the home, rather than having a connection to one another. In the chain of homes where the research was conducted, there had been a recent strategic decision to remove all dedicated activity coordinators from homes so that carers could incorporate activities into their daily workload. However, there was a clear conflict in the provision of autonomy and responsibility for carers to execute tailored activities whilst insisting they follow all processes and systems rigidly.

Cultural norms were also considered as possible external barriers to communication. The very notion of residents being seated in their armchairs for most of the day whilst staff members rushed past them or sat with large folders of paperwork to complete next to them, demonstrated a significant power imbalance and staff superiority. For those with early stages of dementia or big personalities, this arrangement worked well because those residents tended to be noticed by staff, and brief passing conversations occurred. However, for those with disordered language and unaddressed hearing impairment, the task-driven behaviours of staff appeared to create a considerable disconnect between themselves and the residents. So only basic instructions and words were said, and ironically staff spent longer sitting next to these residents in silence to complete their paperwork. The rigid structures in place appeared to leave little time for communication and social interaction. A striking observation from my time in both homes is that staff did not tread lightly in a residents’ home; rather, the residents adapted to the workplace of the staff.

The cultural norms exacerbated the conflicting noise sources in the communal areas of the home. For example, the avoidance of silence in the communal lounges was considered the norm, to make the environment feel lived in and joyful. However, when relatives visited, the excessive noise sources prevented successful conversation from occurring.

### Mechanisms: reduced opportunities for meaningful conversation

The observed internal and external barriers to communication represented the context of both settings, leading to the mechanisms that may be responsible for social isolation. Staff values and preferences were sometimes reported as residents’ values and preferences. Staff mentioned that they would like there to be a cinema room within the home or regular movie afternoons because they felt this would be engaging and enjoyable for residents. However, this would contribute further to the extended period of “sitting and watching” that we observed from most of the resident participants during their waking hours. What’s more, there was a failure to adapt the acoustic environment to suit all residents. During one observation, a participant (Gia) asked a staff member if they could turn the television volume down. The staff member (Lucy) chuckled and said, “if we do that, how will they stay awake?” Gia turned to me and asked if we could go to the library for the interview so that she could “hear herself think”. When it was obvious that residents had hearing difficulties, staff members responded by raising their voices or speaking directly into the residents’ ears. This approach did not appear to enhance the communication between residents and staff members.

The pressures of workload on carers and the displays of productivity directly impacted the time available to communicate with residents. When residents required more time to communicate with people with hearing loss and cognitive decline, this resulted in a tension between communication opportunities and the pressure of daily tasks. The tasks placed on carers were visible to residents. Residents described staff rushing past to perform tasks and completing paperwork in sitting rooms. This was achieved in the presence of residents. A visual busyness resulted in an expectation from residents that staff were too busy to communicate, which led to a perceived power imbalance between the residents and staff. In one interview, a resident was asked:

Researcher: Who do you spend time with here?

Desmond (resident): Myself (laughs) It’s just too difficult to get hold of people. They’ve got other things to do.

One staff member (Ria) commented in an interview:

Look, there are three staff members in the room. I can see they’ve done the folder, and now they’re free to spend time with the residents. What more can we do?

The comment by Ria implied that spending time with residents can only follow administrative tasks such as “doing the folder”.

### Outcome: the effects of social isolation

The context and mechanisms described have led to social isolation as an outcome. Most residents described feelings of hopelessness and undergoing a loss of function. There was a strong sense of what had been lost, as evidenced the interview extract below:

Wendy (resident): “You can’t do what you did before. It’s a very helpless situation. You do what you can, but now you have to wait for others. Nobody can ever do what you do. They can try, but it will never be as good as what you can do for yourself.”

The severity of a resident’s dementia influenced the amount of communication they had with staff members. Inevitably, disordered language becomes more prominent in advanced stages of dementia. Having a hearing impairment, in addition, makes it difficult for someone to talk to, so you are not afforded the same opportunities as somebody else. This was frequently observed in the type of communication and interaction between staff and residents. The following fieldnote extract provides an example of disordered language witnessed by the researcher: “Staff member is helping resident to stand from their seated position. Resident is trying to converse with staff member. They appear to have trouble recalling the staff member’s name and there are long pauses between the resident’s verbal utterances. The resident points to the window and then crossed their arms across their chest to gesture that it is cold outside. The staff member does not appear to notice and ushers the resident towards the dining room. There is a lack of cohesion in the resident’s words and they continue looking out the window as they walk”. It appeared that for those with disordered language, any communication from staff was largely task-focused and centred around the practicalities of personal care and mealtimes. There was no evidence of staff making positive non-verbal communication attempts, which could be due to a lack of effort/willingness or confidence in effective engagement. This is evidenced in one interview with the wife of a resident who has advanced dementia.

Catrina (relative): “I don’t think he’s too happy here…He’s been in this room for seven years. Can’t do anything about it. Doesn’t talk much, doesn’t say much. I don’t like watching him here.”

A lack of value was placed on a meaningful conversation, i.e. topics related to resident values, beliefs, and experiences. This was observed with staff prioritising physical care towards residents and restricting discussion to “small talk”. This was internalised by residents who, in turn, appeared indifferent about communicating with other residents, as evidenced in this fieldnote extract: Lucy assists a resident (Jenny) into a lounge chair; she raises her voice and demonstrates elder speak. “How are you this morning? Okay, get comfortable and stay put; the drinks trolley will be here soon,” she says and walks off without waiting for a response from the resident. Another resident (Chloe) walks past and says good morning, but Jenny sitting down shakes her head and closes her eyes. Therefore the “system” (tasks, routines, processes of the home) appeared to be the primary focus in the two settings, with residents as passive recipients, and staff members as facilitators. The long periods of sitting and waiting observed of residents contributed to a feeling of system dependence and passivity.

The experience of social isolation and the loss of social identity manifested in different ways. One resident appeared depressed with his living situation, which may have contributed to their lack of connectedness and engagement with others, as evidenced in this interview extract:

Researcher: “Do you have friends here in this home?”

Leon (resident): “No…what’s the point? You come here, and suddenly you’re waiting to die.”

Leon spent much of the day sitting alone in the home’s entrance area. Residents or staff members filling out paperwork next to him were not observed to interact, and Leon did not engage either. Leon did not receive any visitors throughout the two-week ethnographic period, and when asked informally if he saw his friends and family, he chuckled and shook his head. The hard floors and high ceilings in the entrance area where he sat meant that sound echoed across the walls, potentially making conversation difficult. Similarly, the layout of the armchairs did not encourage interaction and eye contact.

One resident commented, “my day consists of sitting in a chair, so what have I got to look forward to?” This quote suggests a sense of hopelessness and lack of worth for meaningful relationships. The lack of connection to anyone apart from relatives who may only visit once a fortnight and the lack of connection to the building as their home contributed to experiencing social isolation as an outcome within the context and mechanisms of the care home settings.

The paperwork demands were seen as a necessary priority for staff. Combined with the reported lack of communication training for staff, this left little time and space for them to “read between the lines” of resident utterances. This was observed in residents whose language was disordered and whose hearing difficulties were not addressed via hearing aids or assistive technology. These residents had less chance of positive interactions and social engagement.

### Social interactions

When social engagement did occur, care staff were observed sitting with residents during mealtimes and engaging in conversation. There were also examples of intermittent structured activities with an opportunity for interaction between staff and residents. Staff were clear about the residents they most enjoyed speaking to. Their language was not disordered in every case, and they made active communication and positive utterances toward staff (smiling, waving, and verbal gratitude). Therefore, the team valued these residents and made an active effort to speak and interact with them, especially those residents who showed gratitude and performed in the ‘sick role’. This occurred most often in care home B, where the communal lounge was used as a dedicated space for interaction rather than a walk-through area in care home A. Care home B also had a smaller dining area than care home A, with less ambient noise. This created an optimal environment for conversation between care staff and residents during mealtimes and amongst residents without disordered language.

## Discussion

The multiple methods used within the ethnography methodology provided a holistic view of residential care settings. We have examined the topic of social isolation from an environmental audit, observations, and interview lens, triangulating the data to make sense of the central phenomenon. The realist perspective provided an understanding of the context, mechanisms, and outcomes within the closed system of two residential care settings. Figure 2 explains how and why social isolation occurs in these settings. Social isolation is maintained because of the rigid structure of care systems in prioritising physical care and completing paperwork. When this is combined with the challenges care staff face in understanding residents with disordered language and using the biomedical model to manage hearing loss, the opportunities for meaningful conversation are reduced. Staff members retained a distance between themselves and the residents. This may have been to separate their workplace identities from their social identities. They referred to residents as “customers”, which maintained the distance. There appeared to be separate social groups within the homes: staff members were one group who had a connection to one another, i.e., laughing and joking with one another, and the residents as another group. The latter were disparate individuals who did not have a connection to one another. Therefore, residents employed a passive group role, as determined by the social identity approach, with little opportunity for change. There was no emphasis on social activity or the potential of being an active social group, so the status quo was maintained.

A shift from pathogenic to salutogenic care is required to prioritise communication in these settings [68]. The former refers to dualistic thinking, i.e., categorising a person as either healthy or visibly diseased and prioritising medical treatment. The latter refers to the ability to see the entire person rather than solely the disease. For example, when residents discuss past events in their life (that may be unclear), staff members could sit and listen through the disordered language and engage in meaningful conversation instead of dismissing their words or replying with ‘elder speak’. In other words, both verbal and non-verbal cues from residents are acknowledged. This is a critical factor in reducing isolation within these settings. Where person-centred care and social engagement arose between staff and residents, it was clear that residents valued these interactions, which led to enhanced conversations between residents.

Our study offers novel insight into how and why social isolation occurs in residential care settings compared to previous studies that have focussed on communication alone [20, 23, 42]. The social identity approach to health and wellbeing has given us a broader perspective on the social needs of residents beyond sitting in communal areas or taking part in activities. The nuances and complexities of a residential care home community lend itself well to the social identity approach to health and wellbeing [33]. The findings of our study presented few examples of social groups and interactions between residents. The level of connectedness and engagement experienced by residents could be enhanced or reduced depending on several factors. For example, the acoustic environment of the communal areas was identified as a potential barrier to communication. According to social identity approach [29], this could impact on a resident’s sense of identity. Moreover, when considering connectedness, there was a distinction observed between residents feeling a connection with their physical environment and the familiar routine processes within the home, as opposed to a connection to those around them. This could be because the nature of ‘care’ is pathogenecised rather than salutogenecised [68]. Staff are trained to prioritise a resident’s physical needs over their communicative needs. This results in social engagement between staff and residents that is either overlooked or subject to time constraints. Therefore, those most challenging to communicate with are not given the time or opportunity to be “heard” because of the effort required to read and understand their non-verbal cues and verbal utterances [69]. This limits the opportunity for residents to experience social connectedness and retain their social group identity within the home. This is perpetuated by the lack of choice in residents’ communication options and exacerbates the experience of social isolation. This is due to little control over the acoustic environment and their degree of language impairment dictating how meaningful conversation maybe with other residents and staff. These findings offer similar perspectives to residential care research carried out over ten years ago [20], highlighting the continued issues with ‘care’ provision, even though person-centred care and decision-making now appear on best practice NICE guidance [70]. Bureaucracy has changed practice requiring care staff to fill out vast amounts of paperwork for each resident; not surprisingly, this has led to fewer opportunities for meaningful social engagement with residents. Therefore, financial commitment from the care homes, and changes to staffing levels and skill mix (for example, employing and training activities coordinators, using volunteer visiting schemes, etc.) are tangible changes that could help improve overall social engagement.

The environmental and social factors that can hinder successful communication and person-centred care within residential care could be avoided or resolved [23, 42], whilst acknowledging that the individual preferences of residents could clash. The realities of the physical and social constructs of care homes result in individuals experiencing poor quality of communication due to varying simultaneous noise sources (television, radio, conversations in communal areas etc.) that residents have little control over [20]. Those individuals with hearing impairment may be more vulnerable to social isolation as their difficulties with communication at mealtimes and during other social activities restricts them from wholly participating [71]. It is likely that these individuals do not feel part of a social group within the home and have no reference of “us” and “we” (according to the social identity approach for health and wellbeing) because their communication difficulties isolate them. The limited training of care home staff, ineffective communication [72], and general lack of time and opportunity for engaging with residents [42], means it is often easier for residents to withdraw to their rooms or prioritise non-socially engaging activities such as reading rather than join social activities [24]. The extra effort and ‘work’ required to communicate effectively with people living with hearing loss can result in avoidance of conversation or minimal contact [73]. Where care staff understand a resident’s individualised needs, confusion in communication was generally avoided. This required consideration of the residents’ co-morbidities and preferences and distinguishing between any hearing and cognitive impairment that may be present [74]. Whilst training for care staff exists, it is unlikely to be delivered to a consistent standard and frequency throughout the UK. This may be because of the burden on senior management experience due to high staff turnover [75] or the lack of importance in communication training within this sector. The challenge for care home staff to promote independence and social interaction among older people with dementia and sensory impairments should not be neglected. Effective staff training requires understanding the complexities of sensory impairments, social isolation, and dementia in older adults [76].

Social isolation is one of the most critical factors in the social determinants of healthy ageing [77]. This study has highlighted the value of social connectedness and revealed the need for interventions to be developed that will encourage social engagement and identity within the context of residential care. The humanised elements of care include dedicated attention to communication through meaningful and sincere actions [78], as well as acknowledging and embracing the social roles and groups within residential care settings according to the social identitiy approach to health and wellbing. These are not a priority for staff because their training is focused on routine and bodily tasks. This is a systemic issue within residential care, based on financial structure, high staff turnover, and overlooking of communication as a valuable activity [24]. Those with reasonably good language and communication skills engaged in some level of meaningful and humorous conversation with staff, therefore avoiding a vulnerable state of social isolation. The critical mechanism appears to be the level of expressive and receptive language a person has [79]. This is the deciding factor in whether a care home resident experiences social isolation or not, evidenced by the social engagement that did occur during the study. An example of the type of training that care staff could engage in is a focus toward humanisation and its essential components [80], which could trigger better communication and greater engagement with residents with disordered language. However, this requires adjustments in their perception of time and efficiency to ensure all necessary tasks are still completed.

Previous research has focussed on hearing loss and remediating hearing loss, but this does not appear to influence the quality of relationships [81, 82]. If you’re liked, you’re more likely to have a meaningful conversation, reducing the chances of social isolation. This has been evidenced by the popularity amongst staff of one resident participant with profound hearing loss and early-stage dementia compared to other residents with lesser degrees of hearing impairment but advanced stages of dementia, which are not afforded the same communication opportunities. Whilst the initial PaE phase and environmental audit highlighted obvious environmental factors that contributed to communication barriers, even with these overcome, a systemic change in the type of communication directed toward residents is required. These findings align with parts of the programme theory identified in a realist synthesis, where communication in care homes amongst adults living with hearing loss and dementia was explored [51]. Social integration has been shown to delay memory loss in older adults [83]. Immediate and delayed recall scores declined at half the rate over six years for those with higher social integration at baseline. This provides evidence of the protective effect of social integration on cognitive health outcomes. The social identity to health and wellbeing approach outlines the importance and value of group membership and the ability to have meaningful interactions [28]. This has been shown in the few residents who conversed with the staff in a meaningful and humorous manner and appeared content with their surroundings.

### Limitations

Research observations were limited to communal areas, so it is not known how communication may be handled in smaller spaces, such as the residents’ rooms. Interestingly, staff participants who were housekeepers of the care homes spoke of the daily detailed conversations they have with residents during the 30–60 min they are cleaning their private spaces. Further research exploring communication patterns in these intimate spaces would be necessary to better understand the social isolation phenomenon within these environments. Since dementia and hearing loss are almost ubiquitous in care homes, separating their effects on other variables (such as social isolation) will always be challenging but worthy of exploration.

While the PaE phase involved potential participants’ views on methods, it was impossible to involve those persons in the data analysis and theory emergence due to funding limitations [84] and the Covid-19 pandemic restrictions. Moreover, the exclusion of participants who did not have the capacity to consent limits the applicability of the findings. Future research exploring the lived experience of these individuals would be worthwhile. In addition, the generalisability of the findings is limited to the heterogeneity of the two care home sites.

### Recommendations

This work has identified the potential for social and environmental recommendations implemented within the homes to help improve communication and reduce social isolation. For example, reducing power imbalance could involve staff dining with residents during their mealtimes. This would encourage conversation and allow residents to view staff members as companions who share their home rather than purely caregivers. Furthermore, specialist training from Hearing Therapy and Speech and Language Therapy could provide care staff with valuable skills in enabling meaningful conversation and listening through disordered language. Greater awareness of acoustic factors contributing to reduced communication opportunities must also be considered. In addition, the barriers to accessing Audiology services should also be addressed, which will be specific to each location. These recommendations align with previous research [42] that suggested interventions for enhanced communication opportunities, but our work focuses mainly on overcoming social isolation. Of course, interventions are dependent on fiscal and government systems. Still, there are potentially significant efficiencies in creating a culture conducive to meaningful communication, not only in terms of residents’ quality of life but also in reductions in work-related stress and staff turnover.

### Electronic supplementary material

Below is the link to the electronic supplementary material.

Supplementary Material 1

## Data Availability

The datasets generated and analysed during the current study are not publicly available as this work forms part of a PhD thesis that has not yet been published but are available from the corresponding author on reasonable request.
