# Supplementary material for: An ethnography study exploring factors that influence social isolation in care home residents living with dementia and hearing loss
Source: BMC Geriatr. 2023 Sep 25;23:593. doi: 10.1186/s12877-023-04296-0 (PMC10518931; doi:10.1186/s12877-023-04296-0)
Supplement: Supplementary file 1 — Supplementary Material 1 [file 12877_2023_4296_MOESM1_ESM.docx]

Appendix 1 - Environmental audit proforma used in ethnographic work at care homes A and B

|  | Current Situation | Changes Recommended |
| --- | --- | --- |
| Is there a sound field and/or loop in the room? |  |  |
| Are hearing maintenance kits and schedules easily available? |  |  |
| Are there quiet rooms or areas for relaxing? |  |  |
| Is there accessible information about the needs of individuals with hearing impairment? |  |  |
| Is there an echo when a sound is made? |  |  |
| Are there soft furnishings in the room? |  |  |
| Are there acoustic ceiling or wall tiles? |  |  |
| Are tables, shelving, storage covered to reduce noise? |  |  |
| Are there carpets in the room or is flooring low echo? |  |  |
| Are there curtains or blinds? |  |  |
| Do doors fit well and have an acoustic seal? |  |  |
| Is there double/triple or single glazing? |  |  |
| Are ceilings high or low? |  |  |
| Do all chairs and tables have rubber feet? Are they intact? |  |  |
| What sounds are in the communal and dining rooms? |  |  |
| What sounds are coming from outside the communal areas? |  |  |
| What sounds are coming from outside the home? |  |  |
